# Glass Fibre-Reinforced Extrusion 3D-Printed Composites: Experimental and Numerical Study of Mechanical Properties

**DOI:** 10.3390/polym16020212

**Published:** 2024-01-11

**Authors:** András Kámán, László Balogh, Bálint Levente Tarcsay, Miklós Jakab, Armand Meszlényi, Tamás Turcsán, Attila Egedy

**Affiliations:** 1 Department of Process Engineering, Faculty of Engineering, University of Pannonia, H-8200 Veszprém, Hungary; kaman.andras@mk.uni-pannon.hu (A.K.); balogh.laszlo@mk.uni-pannon.hu (L.B.); tarcsay.balint@mk.uni-pannon.hu (B.L.T.); meszlenyi.armand@mk.uni-pannon.hu (A.M.); 2 Department of Material Sciences, Faculty of Engineering, University of Pannonia, H-8200 Veszprém, Hungary; jakab.miklos@mk.uni-pannon.hu; 3 eCon Engineering Kft., H-1116 Budapest, Hungary; tamas.turcsan@econengineering.com

**Keywords:** 3D printing, additive manufacturing, nylon composites, tensile strength, Young’s modulus, process parameters

## Abstract

The properties of 3D-printed bodies are an essential part of both the industrial and research sectors, as the manufacturers try to improve them in order to make this now additive manufacturing method more appealing compared to conventional manufacturing methods, like injection moulding. Great achievements were accomplished in both 3D printing materials and machines that made 3D printing a viable way to produce parts in recent years. However, in terms of printing parameters, there is still much room for advancements. This paper discusses four of the 3D printing parameters that affect the properties of the final products made by chopped glass fibre-filled nylon filaments; these parameters are the printing temperature, nozzle diameter, layer height, and infill orientation. Furthermore, a polynomial function was fitted to the measured data points, which made it possible to calculate the tensile strength, flexural strength, and Young’s modulus of the 3D-printed samples based on their printing parameters. A Pearson correlation analysis was also carried out to determine the impact of each parameter on all three mechanical properties studied. Both the infill orientation and printing temperature had a significant effect on both strengths and Young’s modulus, while the effect of nozzle diameters and layer heights were dependent on the infill orientation used. Also, a model with excellent performance was established to predict the three mechanical properties of the samples based on the four major parameters used. As expected from a fibre-reinforced material, the infill orientation had the most significant effect on the tensile strength, flexural strength, and Young’s modulus. The temperature was also quite significant, while the nozzle diameters and layer height effect were situational. The highest values for the tensile strength, flexural strength, and Young’s modulus were 72 MPa, 78.63 MPa, and 4243 MPa, respectively, which are around the same values the manufacturer states.

## 1. Introduction

In recent years, the 3D printing industry had explosive growth in both household and industrial applications. Depending on the printing parameters, the properties of the end product can vary greatly. So, in order to determine the parameters to the demands of different applications, the effects of individual parameters must be studied. Numerous studies that discuss different parameters’ effects on the 3D printing process and end product can be found. However, due to the rapid development in this area, most of these studies are either out of date or the used 3D printer, slicing software, firmware, and materials are not consistent or lack the adjustability and sufficient documentation in order to reproduce the researched results. The aim of this study is to determine the effect and impact of the key parameters used during a printing process on the properties of the final product. These key parameters are the printing temperature, layer height, nozzle diameter, and infill orientation. The three mechanical properties of the end products, which were measured during testing, are the tensile strength, flexural strength, and Young’s modulus. Most of these parameters and properties are common when a study is in the field of additive manufacturing. Muhammad et al. investigated the effects of eight parameters that could change the properties of PLA 3D-printed products [1]. These were the layer height, perimeter line count, infill density, infill angle, printing speed, printing temperature, bed temperature, and print orientation. There are parameters that cannot be modified due to the material used, the geometry of the body, or the printing hardware, and therefore, the investigation of such parameters is questionable; for example, the temperature of the build plate is usually chosen based on the material in order to ensure the adhesion of the printed body and the threshold in which the adhesion is adequate is quite narrow. The effect of the parameters changes when fibres are added to the materials [2]; therefore, composited and unreinforced plastic filaments should be studied separately. Also, because of the added composite particles, the effect of a single parameter could also vary at different sizes [3], and because these particles cannot be compressed, the lower end of the used nozzle diameter is limited. Nylon is one of the most widely used polymers worldwide; it is a thermoplastic that has great mechanical properties and a low price, which is frequently used with some kind of fibre reinforcement. Due to its high strength, impact resistance, UV and chemical resistance, lightweight nature, and durability, nylon can be used in various applications, such as clothing or paracord manufacturing as a fibre; as a lightweight, high-strength, and durable solid plastic in small arms and machine parts manufacturing; or in the electronics sector, to name a few. Their reinforcement is usually conducted by mixing chopped glass or carbon fibres into the plastic matrix [4,5,6], which oftentimes makes the orientation of said fibres random, especially when one of the most common manufacturing methods, injection moulding, is used; the orientation and distribution of fibres inside the injection-moulded chopped fibre-reinforced plastic bodies are challenging to change from the random nature that the flow of plastic inside moulds provides [7,8,9]. However, during an extrusion 3D printing process, the fibres inside the molten plastic naturally orient themselves in the direction of the extrusion [10,11,12,13], so the desired orientation can be achieved more easily. One of the most used fibre-reinforced composite filaments in functional 3D printing is the chopped glass or carbon fibre-reinforced nylon filaments due to their mechanical, chemical, and thermal properties [14]. Research on the effect of the infill of such materials can already be found [15,16,17,18], mainly due to the fact that the orientation of fibres inside the nylon should contribute to its mechanical properties significantly, and the most investigated mechanical properties are the tensile and compressive strengths of the samples, which is quite strange considering the fact that fibre reinforcement is commonly used to enhance materials’ flexural strength. However, these studies are quite limited in their scope and do not investigate other important parameters, and the choice of other parameters is hardly stated, and often when they are stated, they seem to be chosen randomly or are the factory presets, which should be modified based on a number of factors. Furthermore, the almost total lack of information on printing conditions often makes it difficult to replicate the results obtained in said studies and the use of low-end commercial 3D printers can be quite limiting in terms of parameter thresholds (e.g., a PTFE tube cannot withstand temperatures above 240–250 °C without severe degradation, which makes the likes of nylon filaments not able to be printed at higher temperatures), usable hotend and nozzle types, and advanced features, which are crucial for calibration. Aside from these deficiencies, studies investigating this field of technology are few and far between, making the citation of such studies quite challenging. Therefore, in this study, all the used printing parameters (over 450), chief 3D printer components, and printing conditions are available. So, in order to ensure the repeatability of the study and make the conditions more clear, the full list of parameters is included. Also, to try to alleviate the problematic aspect of imperfections during 3D printing, extensive testing and calibration of both the printer and material were carried out. Lastly, the 3D printers used in studies fall between two groups, the industrial and semi-industrial printers, which are great for production purposes but are quite lacking in terms of customization, adjustability of the parameters, and usability of filaments that are not from the manufacturer. The other group is the cheap commercial printers, which have the aforementioned qualities but lack quality parts and usually run on some type of Marlin firmware, which lacks the advanced features that other firmware provides. These are the reasons that, in this study, one of the highest quality hobbyist 3D printers was used with the most recent features that can be found in any firmware.

## 2. Materials and Methods

**Figure 1:**
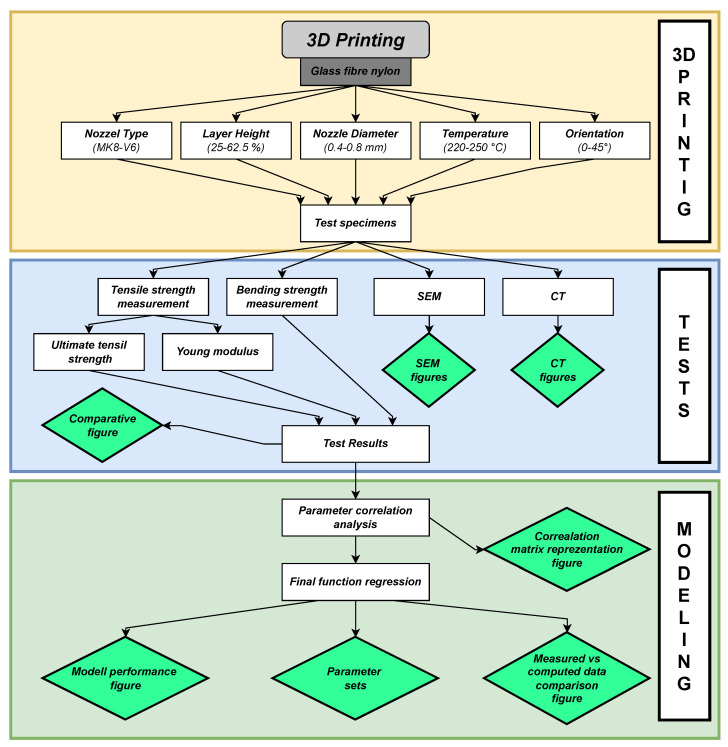
Summarization of the tests and methods used during the research phase.

The investigated parameters, measured properties, as well as the analysis methods used during this study can be observed in their entirety in Figure 1.

### 2.1. Materials and 3D Printer Used

Repeatability is an essential aim of this study; the full list of components used during printing as well as the processes before and during printing are stated below.

The 3D-printed samples were printed on a Rat Rig V-Core 3 (Loulé, Portugal), which is a modular CoreXY FDM 3D printer that uses Klipper firmware and features quality mechanical parts. For precise plastic deposition, an LDO Orbiter 1.5 extruder (Shenzhen, China) and Slice Engineering Mosquito hotend (Gainesville, FL, USA) were used. Klipper firmware (v.0.11.0) has advanced features that greatly enhance 3D-printed objects’ quality, for example, pressure advance, which calculates the amount of deposited material based on the hotend pressure [19], rather than the filament fed into the hotend, and skew correction, which compensates for the geometric distortion of the 3D printer frame and ensures the dimensional accuracy [20]. Prior to printing the samples, the advanced features and basic settings were calibrated. The G-code for each sample was generated in the SuperSlicer software (v.2.5.59). Due to the number of parameters that can be adjusted in it, this paper only mentions the main ones, but the complete list is included in Appendix A. During the printing, the cooling was disabled for better bonding between the layers, and because of this, the printing speed had to be reduced as well; it was 40 mm/s as default. In this study, the used 3D printing material was a single roll of nylon 6/66 (as a mixture of nylon 6 and nylon 66 polymers, as stated by the manufacturer) filament reinforced with 25 wt% chopped glass fibre of 1.75 mm diameter. It was manufactured by Shenzhen Esun Industrial Co., Ltd. (Shenzhen, China), which has a reputation for its affordable and high-quality 3D printing filaments. Due to the hydrophilic nature of nylon filaments, the chopped glass fibre-reinforced nylon filament was dried at 70 °C for 24 h before printing and was under constant drying while the printing took place. The test was conducted on an INSTRON 3400 series universal testing machine (250 kN) (Norwood, MA, USA). The specimens were an ASTM D638 type V due to their smaller size. In this study, each sample represents four parallel measurements, whose values were filtered based on deviation.

### 2.2. Investigated Parameters

Previous tests showed that the behaviour of the used materials was more predictable when printing was performed with no wall, top or bottom solid layers, and infill only due to the more homogeneous nature of end products. In real-world applications, horizontal and vertical perimeters (walls) must be used to ensure the objects’ dimensional accuracy. However, from an investigation standpoint, the perimeters of a 3D-printed object make it more difficult to measure the impacts of the parameters, so the wall line count parameter was removed. The investigated four parameters were the printing temperature (temperature of the printing head), nozzle diameter, layer height, and infill orientation. Furthermore, the nozzle diameter and layer height were later merged into a single virtual parameter as a way to measure the amount of material extruded in a single line. All the parameters used can be observed in the config text file in Appendix A, which contains more than 450 parameters used during printing but not changed except for the 4 investigated parameters.

### 2.3. Nozzle Diameter

**Figure 2:**
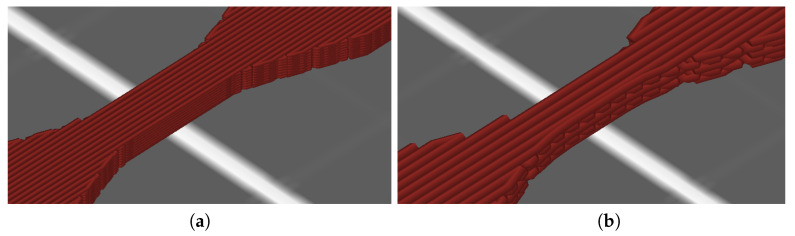
The difference between the extruded lines of (**a**) 0.4 mm nozzle diameter and (**b**) 0.8 mm nozzle diameter.

The nozzle diameter is the only hardware-related parameter and can only vary based on the type of nozzle used. It is a deterministic dimensional parameter that mainly affects the amount of material that can be extruded and the amount of extruded lines a body is made of across the XY plane (Figure 2).

### 2.4. Layer Height

Similar to the nozzle diameter, the layer height is a dimensional parameter and mainly affects the geometry of extruded lines. The lower end of the layer heights corresponds to a flat extruded line, while the higher end of it means an extruded line similar to the geometry of a squashed circle.

### 2.5. Extruded Line cross-Section Area

**Figure 3:**
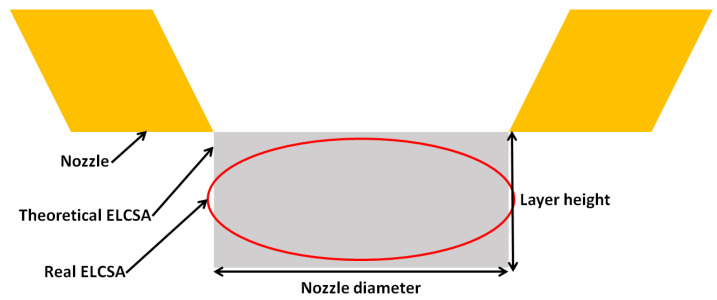
The correlation between nozzle diameter, layer height, and real and theoretical ELCSA.

**Figure 4:**
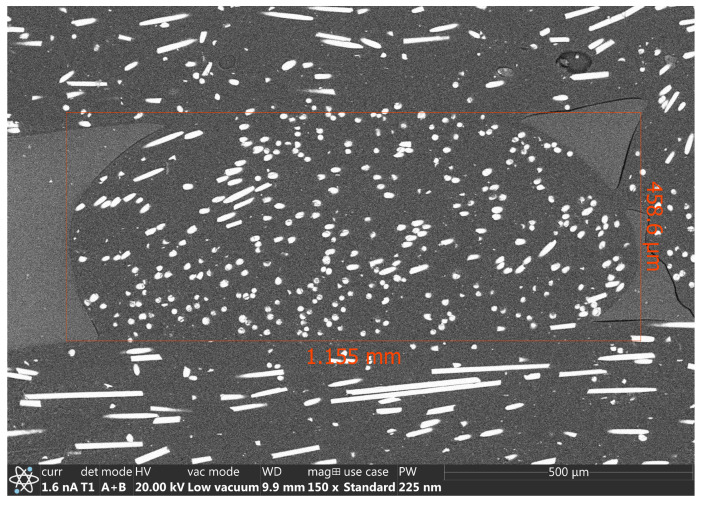
SEM image of ELCSA inside a 3D-printed layer.

During the evaluation of the test results, the problem arose that the nozzle diameter determines the usable layer height, and therefore, these two parameters are not independent of each other. In order to ensure the independence of the investigated parameters, the nozzle diameter and layer height were incorporated into a new theoretical parameter called the extruded line cross-section area or ELCSA, which is the product of the nozzle diameter and layer height (Figure 3 shows the representation of the ELCSA as a schematic image and Figure 4 shows ELCSA as a SEM image). The ELCSA shows the amount of material extruded in a single line.

### 2.6. Printing Temperature

One of the most impactful factors in the final properties of 3D printing objects is the temperature, which contributes to the bonding of extruded lines. There is a general rule in FDM/FFF 3D printing that the lower end of the printing temperature range produces better aesthetic features but weaker mechanical properties, and the opposite is true for the higher end of the printing temperature range. The used temperature is mostly determined by the material extruded; in the case of nylon 6/66, the usual printing temperature range is between 220 and 260 °C, and when they are reinforced with fibres, the temperature tends to be on the higher end. The printing speed or volumetric speed depends greatly on the temperature used because, to achieve higher printing and volumetric speed, the temperature also has to be higher, so the used default and maximum printing speed was 40 $\frac{\mathrm{mm}}{s}$ and the maximum volumetric speed was 12 $\frac{{\mathrm{mm}}^{3}}{s}$ to ensure sufficient bonding even in lower temperatures.

### 2.7. Infill Orientation

Also known as the raster angle in other slicer software, it determines the orientation of the parallel infill lines inside the 3D-printed bodies. In the case of this study, 100% infill was used exclusively in order to ensure the homogeneity of the samples. In the case of 100% infill, the bodies build up from layers, and the layers build up from parallel extruded lines; these layers are stacked on top of each other perpendicular to the one below them. So, an infill orientation of 0° also means an orientation of +90°; due to this fact, the infill orientation can only be varied between 0° and 45°, where the 0° means that half of the number of layers (inside them parallel lines) rounded up are parallel to the axis of the samples and half of the number of layers rounded down are perpendicular to the axis of the samples, and 45° means that all of the layers and the extruded lines inside them are in a 45° orientation to the axis of the samples. The unevenness of the layer counts means that if the number of layers is not an even number, the result of the 0° and 90° infill orientation could differ, but in most of the real-world applications, its significance is so low that it makes no sense to investigate this disparity.

### 2.8. Nozzle Types

**Figure 5:**
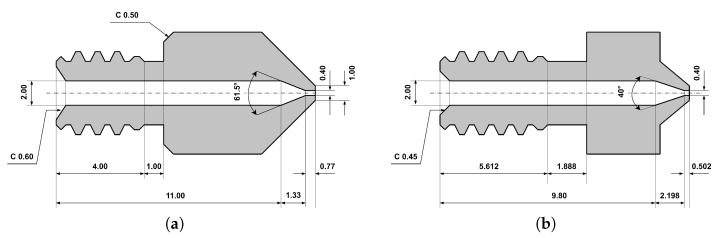
The difference between the (**a**) MK8 nozzle and (**b**) V6 nozzle.

During testing, two types of nozzles were studied. The first was the recommended V6-style nozzle compatible with the Slice Engineering Mosquito hotend and the other was the widely available MK8 nozzle, which, in theory, is too short (5 mm neck size instead of the V6’s 7.5 mm) for the hotend used and a small gap between the bi-metal heatbreak and nozzle can be observed (Figure 5). However, the samples printed with this shorter MK8 nozzle did not show any flaws, so their usage was also considered. Due to the fact that two nozzle types and different horizontal and vertical perimeter counts were studied and showed flawed behaviour, the sample number starts from 35 in this study, which means that there were 34 probing test sample groups and 16 final sample groups, which can be observed in the specimen table included in the Supplementary File.

### 2.9. Instruments

The tensile and flexural strength tests were conducted on an INSTRON 3400 series universal testing machine (250 kN) with a distance between the clamps of 40 mm and an extension rate of 2 mm/s. The specimens were an ASTM D638 type V due to their smaller size. The investigation of the different characteristics of the fracture surfaces was conducted by FEI/Thermofischer Apreo S; Philips XL 30 ESEM scanning electron microscopy (SEM, Brno, Czech Republic) and Nikon XT H 225 ST X-ray computed tomography (CT, Tokyo, Japan). The porosity and fibre orientation analyses were performed with CT.

### 2.10. Scanning Electron Microscopy

**Figure 6:**
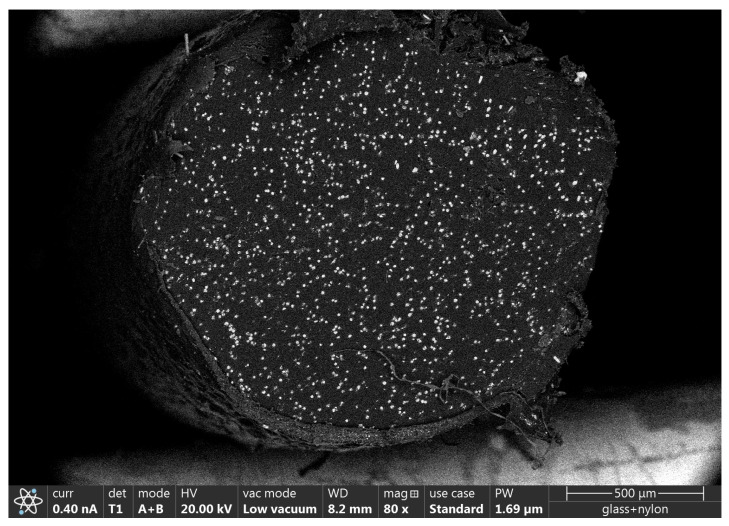
SEM image of the distribution of the chopped glass fibres inside the filament.

The length of the chopped glass fibres was measured with SEM; prior to it, the nylon matrix of the reinforced filament was burnt away, so the individual glass fibres were exposed entirely. The width of the fibres varied between 10 and 15 µm, and their length could reach up to 475 µm, which is not only comparable to the diameter of the nozzle used (400 µm) but exceeds it. A distribution of the fibres across the cross-section of the filament can be seen in Figure 6.

### 2.11. Computed Tomography

**Figure 7:**
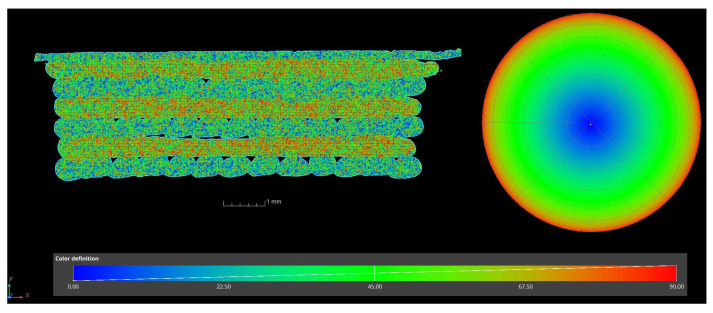
The orientation of fibres inside individual extruded lines at a cross-section of a sample.

The sample’s porosity and fibre orientation were analysed with computed tomography. The fibres are oriented in the direction of extrusion by the flow of the polymer, meaning that the orientation of fibres can be adjusted with infill orientation. Figure 7 shows the orientation map of the fibres inside individual extruded lines, where red means the parallel orientation of fibres with the plane of the image, blue means a perpendicular orientation of fibres with the plane of the image, and green means an orientation between the two. The mostly blue and green parts are cross-sections of multiple extruded lines, while the mostly red and green parts are along the axis of three separate extruded lines.

**Figure 8:**
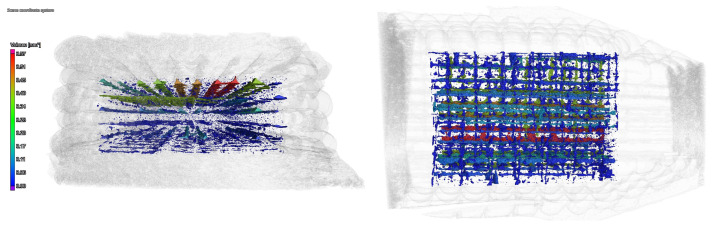
CT image of the porosity system of a 3D-printed sample.

The porosity of particular samples was also measured with computed tomography; these showed a well-defined porosity system built up from the cavities that can be found between extruded lines and follow the direction of the line (Figure 8).

## 3. Results and Discussion

Because of the complexity of the mechanical property figures, the deviation of the values is presented in Table 1 along with the values of the samples printed with MK8-type nozzles.

### 3.1. Tensile Strength

**Figure 9:**
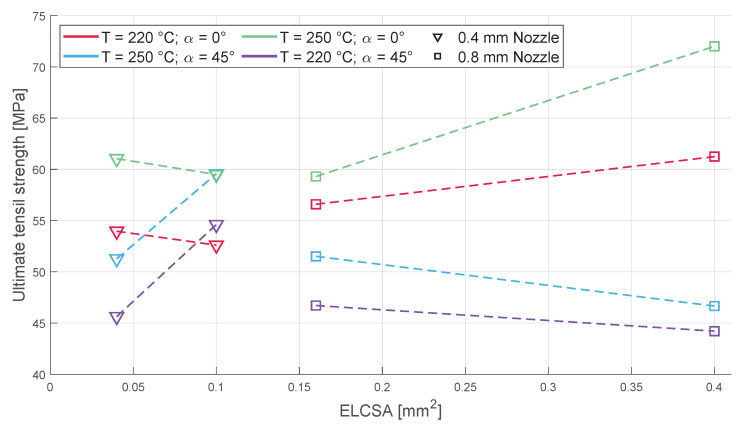
The change in tensile strength based on different parameters.

**Figure 10:**
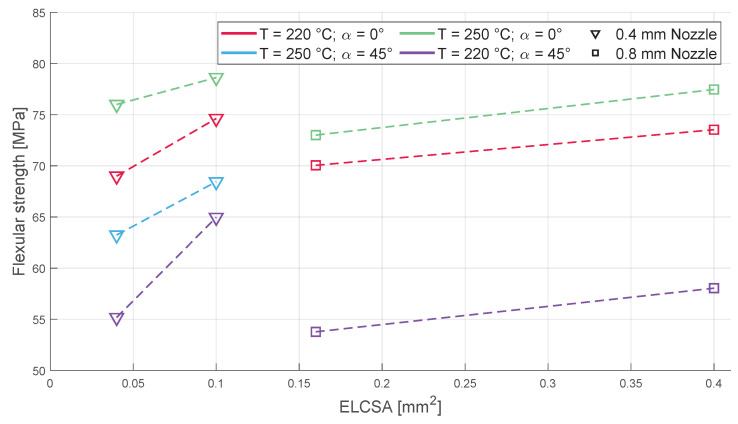
The change in flexural strength based on different parameters: on the left side, the two layer height used with the 0.4 mm diameter nozzle and, on the right side, the two layer height used with the 0.8 mm diameter nozzle can be seen linked together.

As one of the most important mechanical properties of plastics, the tensile strength tests were carried out first. The 0° and 45° infill orientations behaved differently (Figure 9); for example, the increase in layer height had the opposite effect on the two orientations mentioned, so in order to gain any usable information from the tensile tests, the evaluation of these tests was performed separately. Furthermore, the infill orientation had the most impact on the tensile strength of the specimens, not surprisingly due to the nature of the fibre reinforcement. The behaviour of the samples with a 45° infill orientation (when all of the extruded fibres are in the orientation of 45° to the tensile testing direction) and a 0.4 mm nozzle diameter is strangely similar to the behaviour of the flexural strength samples that can be seen in Figure 10 when we consider that the glass fibres, which can reach up to 0.47 mm in length and the used nozzle is 0.4 mm in diameter, can be longer than the extruded line in which they are composited in. The possibility that these fibres cannot orient themselves in the direction of the tensile stress during testing and therefore suffer flexural stress may be an answer for the similar behaviour shown. This phenomenon was not present when the nozzle diameter was larger (0.8 mm), and the tensile strength lowered with the increase in the layer height. The opposite was true for the 0° infill orientation (when half of the extruded fibres are in an orientation of 0° and the other half in an orientation of 90° to the tensile testing direction) when the 0.4 mm diameter nozzle was used; the flatter extruded lines (low layer height) yielded better tensile strengths, while in the case of the 0.8 mm nozzle, the flatter extruded lines yielded worse tensile strengths. ELCSA, which shows the amount of material extruded in a single line, had an inconsequential effect on the overall tensile strength of the samples as the behaviour of the different nozzle sizes and layer heights varied drastically. The effect of the printing temperature paints a much clearer picture, as the higher temperature always resulted in better strength during both the tensile and flexural testing.

### 3.2. Flexural Strength

The behaviour of all the sample groups was identical during the flexural tests (Figure 10). The infill orientation again had the most impact on the flexural strength of the samples; all the 0°-infill-oriented samples showed higher flexural strength than their 45°-infill-oriented counterparts. The nozzle diameter had a negative effect on the sample’s flexural strengths, which again shows that the amount of material extruded in a single line does not directly contribute to the tensile and flexural strengths of printed bodies. The layer height, however, has a positive impact on the flexural strength, which is not surprising when one considers the fact that the higher layer height makes the extruded lines much thicker in the direction of the flexural stress. Unfortunately, the 250 °C, 45°-infill-oriented, and 0.8 mm nozzle samples were not measurable due to a printing failure (and the intention of using a single spool of filament meant that further printing was not feasible); however, despite their absence, the flexural strength of the remaining samples shows a clear tendency based on the parameters used.

### 3.3. Young’s Modulus

**Figure 11:**
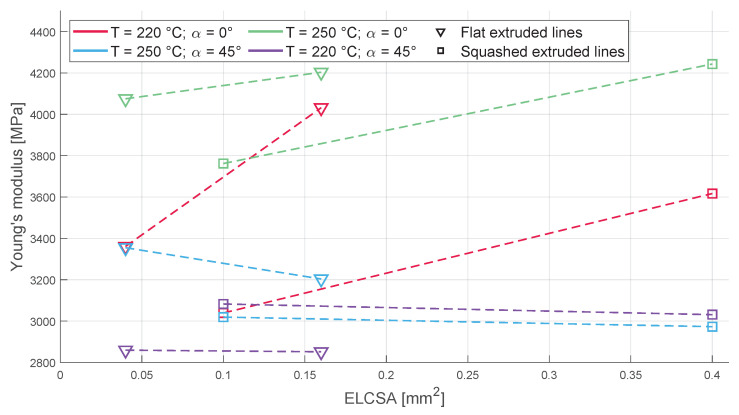
The change in Young’s modulus based on different parameters: on the left side, the two nozzle diameters used with a flat extruded line and, on the right side, the two nozzle diameters used with the more circular extruded line can be seen linked together.

Again the behaviour of the 0°- and 45°-infill-oriented samples differ with the change in the layer height (Figure 11). The increase in the layer height increases Young’s modulus of the 0°-infill-oriented samples and decreases Young’s modulus of the 45°-oriented ones slightly, and with the increase in the nozzle diameter, Young’s modulus decreases. The higher temperature samples had a higher Young’s modulus compared to their lower temperature counterparts. ELCSA again had no impact on the Young’s modulus of the samples.

## 4. Modelling

**Figure 12:**
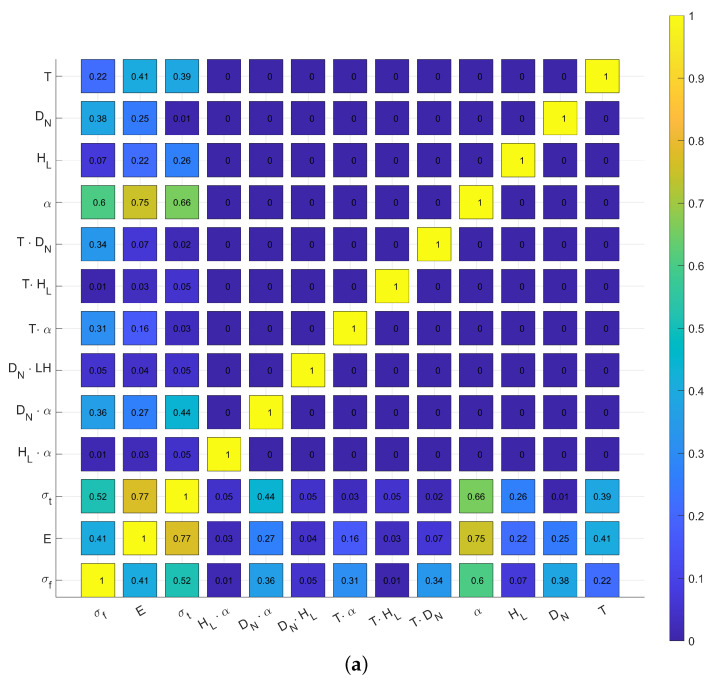

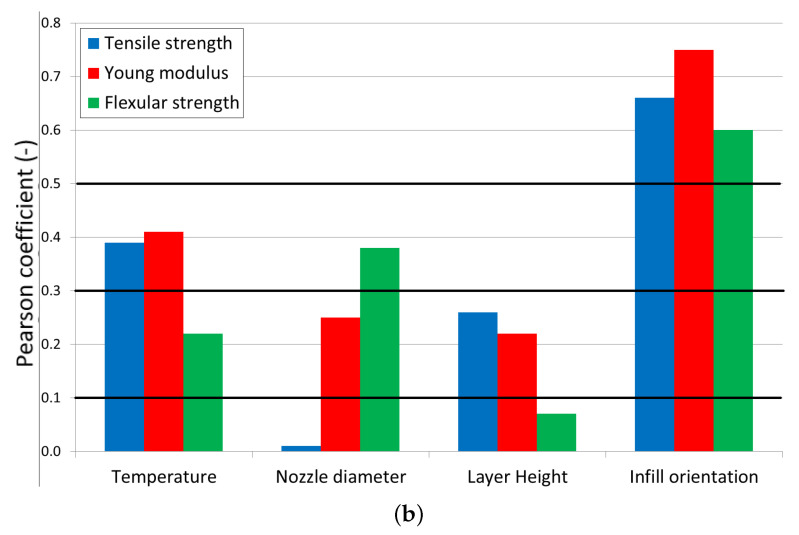
(**a**) The Pearson correlation matrix regarding printing parameters and mechanical properties of samples and (**b**) the summarization of the Pearson coefficient values of the same matrix.

Firstly, to estimate the correlation between each parameter and the mechanical properties, a Pearson correlation analysis was carried out, Equation (1). Also, the values of all the parameters were normalised between −1 and 1, where −1 is the lower end and 1 is the higher end of the parameters, in order to ensure that their original numerical values do not skew the correlation analysis. The results of the correlation analysis and the summarization of these results can be seen in Figure 12. Generally, the corresponding thresholds for these Pearson coefficients are the following:

- No correlation between values of 0 and 0.1;
- A low correlation between values of 0.1 and 0.3;
- A medium correlation between values of 0.3 and 0.5;
- A strong correlation between values of 0.5 and 1.

The infill orientation ( α ) had by far the strongest correlation with all three mechanical properties, which is not surprising considering the nature of fibre-reinforced materials. The temperature (*T*) had a moderate correlation with the tensile strength ( $\sigma_{t}$ ) and Young’s modulus (*E*), which was already apparent in Figure 9, Figure 10 and Figure 11, where all the higher temperature samples had a higher value compared to their lower temperature counterparts. Also, the temperature had a weak correlation with the samples’ flexural strength ( $\sigma_{f}$ ). The correlation between the nozzle diameter ( $N_{D}$ )/layer height ( $L_{H}$ ), both of which are dimensional parameters, and the mechanical properties are not as obvious. The nozzle diameter had a moderate correlation with the flexural strength and showed a low-to-moderate correlation with Young’s modulus comparable to the layer heights; all the while, its correlation with the tensile strength was virtually non-existent. On the other hand, the layer height had a low-to-moderate correlation with the tensile strength and Young’s modulus while having a very low correlation with the flexural strength.

Pearson correlation analysis:

The Pearson correlation analysis measures a linear correlation between variables divided by the product of their standard deviations. In our case, we want to show the effects of the printing parameters on the mechanical properties of the printed material. So, we made the analysis to the unique parameters and the combinations of the parameters so we can get a complete picture of the effects of the parameters. Equation (1) shows the calculation of the Pearson coefficient ( ρ ) for general variables ( $P_{i},P_{j}$ ).

(1) $$\rho \left(P_{i},P_{j}\right)=\frac{\mathrm{cov}\left(P_{i},P_{j}\right)}{\omega_{P_{i}}\omega_{P_{j}}}=\frac{1}{N-1}\sum_{k=1}^{N}\left(\frac{P_{i,k}-\mu_{i}}{\omega_{P_{i}}}\right)\left(\frac{P_{j,k}-\mu_{P_{j}}}{\omega_{P_{j}}}\right)$$

If we made the calculation of every variable, we can obtain, as a result, a matrix ( Φ ) that contains the Pearson coefficient for every variable. That can be represented by the following equation (Equation (2)) for the two general variables.

(2) $$\Phi =\left(\begin{array}{cc} 1 & \rho (P_{i},P_{j})\\ \rho (P_{j},P_{i}) & 1 \end{array}\right)$$

Based on this, we can calculate this matrix for our data; the result can be seen in Figure 12, and the corresponding 95% confidence interval matrix based on the Fisher distribution can be found in Appendix A.

**Figure 13:**
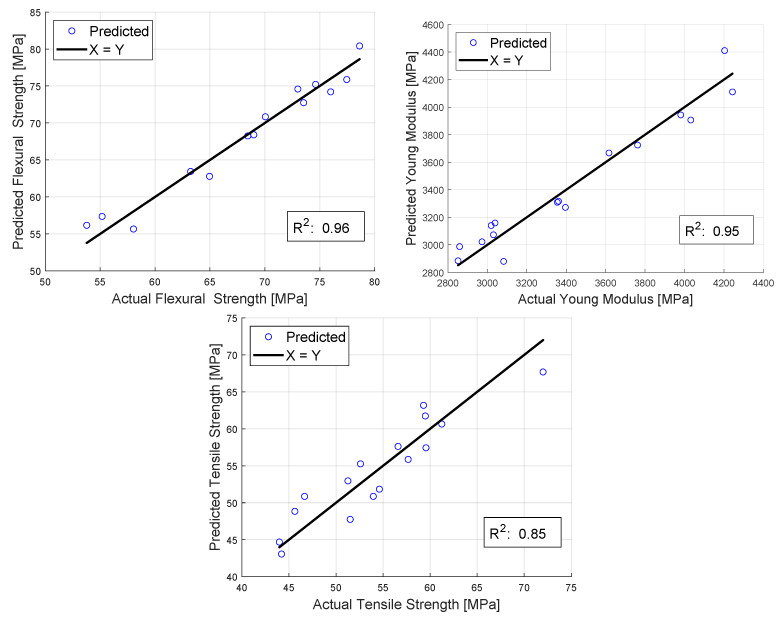
Performance of the model used for all three mechanical properties studied.

When choosing the model for the prediction of the mechanical properties based on the parameters, Equation (3) was chosen. The low correlation between the nozzle diameter and tensile strength, layer height, and flexural strength made a multi-equation model to be considered, but in order to make the working model adequate for all mechanical properties, this idea was dropped. As seen in Figure 13, the performance of the model was excellent for the flexural strength and Young’s modulus while still being good for the tensile strength.

For the approximation of the measured data, we have chosen a polynomial function with the following form (Equation (3)).

(3) $$\Gamma =a+bD_{N}+cT+d\alpha +eH_{L}+fD_{N}T+gD_{N}\alpha +hD_{N}H_{L}+jT\alpha +kTH_{L}+lH_{L}\alpha$$

We only have the data for two points for each investigated characteristic, so the correlation contains only first-order, cross-effect, and bias terms. The function makes a connection between the nozzle diameter ( $D_{N}$ ), the printing temperature (*T*), the layer height ( $H_{L}$ ), and the infill orientation ( α ). For output, we have three examined properties, so the Γ symbol may be Young’s modulus, the tensile strength, and the flexural strength. For the determination of the parameters of Equation (3), we need to solve an extreme value search, which can be formalized in the following form (Equation (4)).

(4) $$\min_{\Omega }\sum_{i=1}^{n}{\left(\Gamma_{\mathrm{comp},i}-\Gamma_{\mathrm{meas},i}\right)}^{2}$$

$$\left[a,b,\cdots ,l\right]\in \Omega$$

The solution to the minimization problem was made in the MATLAB environment, with the least square algorithm. The performance of the model fitting can be characterized with the coefficient of determination ( $R^{2}$ ), which can be formalized with the following equation (Equation (5)), when ${\bar{\Gamma }}_{\mathrm{meas}}$ symbolized the mean of the measured data.

(5) $$R^{2}=\frac{\sum_{i}^{n}{\left(\Gamma_{\mathrm{meas},i}-\Gamma_{\mathrm{comp},i}\right)}^{2}}{\sum_{i}^{n}{\left(\Gamma_{\mathrm{meas},i}-{\bar{\Gamma }}_{\mathrm{meas}}\right)}^{2}}$$

The performance of the model is based on Figure 13. It has satisfactory accuracy, which can be seen also based on the $R^{2}$ values. The best fitting is in the case of the actual flexural strength, and the tensile strength is the worst. Table 2 contains the values of the fitting parameters. The measured and computed values can be seen side by side in Appendix A for all three mechanical properties.

### The Effect of Studied Parameters

To measure the impact of temperature, the previously discussed model was used. Figure 9 and Figure 10 show that the tensile and flexural strength is greatly affected by the temperature used during printing; the higher the temperature, the higher the strength. On the other hand, Young’s modulus lowers as the temperature increases. The impact factor of the temperature can be observed in Figure 12. Also, the different parameter groups showed the exact same behaviour, so the universal conclusion can be drawn that higher temperatures make for better mechanical properties.

Arguably the least discussed printing parameter out of the studied parameters, the tests show that the infill orientation can change the effect of other parameters, such as the layer height or nozzle diameter (Figure 9). Not surprisingly, considering the fact that fibre-reinforced material was used, the results show that the infill orientation was more than twice as impactful as the temperature on the mechanical properties of the samples.

For both the flexural strength and Young’s modulus, the nozzle diameter shows a clear negative effect, while for the tensile strength, it is based on the infill orientation used as the 0°-infill-oriented samples showed greater tensile strengths when the bigger nozzle was used, while the 45°-oriented ones showed the exact opposite. These results contradict the popular belief mentioned previously that a bigger nozzle used during printing makes the printed bodies stronger.

Similarly to the nozzle diameter, the effect of the layer height is clearly positive on the flexural strength of the printed bodies. However, its effect varied, again based on the infill orientation, on Young’s modulus of the samples and also based on the nozzle diameter when the tensile strength is concerned.

Because ELCSA incorporates both the nozzle diameter and layer, both of which show different effects in different cases, the fact that it showed no correlation with any of the mechanical properties is not a surprise. So, the chosen values for the nozzle diameter and layer height should be based on the characteristics and application of the final product (geometry, size, type of stresses, type of failure desired, etc.).

As previously discussed, the use of MK8-type nozzles in V6-compatible hotends is not recommended. However, the availability of the MK8 made the use of it worth considering. The printed bodies showed no flaw, being identical to the V6 nozzle-printed ones at first glance; however, their strength had a significant reduction compared to their counterparts printed with a V6 nozzle, so their further use was dropped and is not included in this study.

## 5. Conclusions

In this paper, the effect of four of the most important parameters used during an FDM/FFF 3D printing process on the three main mechanical properties of glass fibre-reinforced nylon 6/66 filaments was studied, with an additional virtual parameter showing the amount of material extruded in a single line. Also, with a Pearson correlation analysis, the correlation between the parameters and mechanical properties was carried out, and a model capable of predicting the mechanical properties of these glass fibre-reinforced nylon samples with precision was established. The performance of the model for the tensile strength, Young’s modulus, and flexural strength of the samples was 0.85, 0.95, and 0.96, respectively. The infill orientation had by far the most influence on the final mechanical properties. The samples showed greater strength in the direction of the extruded lines, which are stacked on top of each other perpendicularly. This means that the samples were stronger in the infill orientation direction and the direction perpendicular to it, meaning, in this case, a 0° infill orientation, an observation made with carbon fibre-filled and -reinforced nylon-based 3D-printed bodies as well [12,21,22,23]. When future stresses that will play a major role in the failure of a part are known, the infill orientation should be oriented so that the main stress aligns with it [24]. The temperature showed a clear picture, where higher temperatures always resulted in the increase in all three mechanical properties; so, when strength is concerned, the printing of the material should be as high as possible. In the case of the dimensional parameters, when the particular stress types are not known, both should be chosen appropriately based on the final product’s size, geometry, aesthetic, and printing time. However, when the flexural strength is a priority, the nozzle diameter should be as big as possible, and in the case of tensile strength, the layer height should be as high as possible, usually meaning 2/3 to 3/4 of the size of the chosen nozzle.

The highest-value samples in all three mechanical properties were printed with the following:

- A 0° infill orientation;
- A high temperature;
- A high layer height.

The diameter of the nozzle did not have a universal effect on all three mechanical parameters. However, in practice, the diameter of the nozzle is determined by the size and shape of the 3D object or the time of the printing. So, in order to obtain the best possible mechanical properties, the use of an infill orientation in the direction of the stress should be used with a high printing temperature and a layer height that is 3/4 of the appropriate nozzle diameter.

## Figures and Tables

**Table 1:** The measured mechanical properties and used parameters for every sample.

Sample No. (−)	Nozzle Diameter (mm)	Layer Height (mm)	Tempera Ture (°C)	Infill Orientation (°)	Tensile Strength (MPa)		Young’s Modulus (MPa)		Flexural Strength (MPa)	
					Average	Deviation	Average	Deviation	Average	Deviation
1	0.6	0.4	220	45	44.85	6.27	3322	906.03	-	-
2	0.6	0.4	250	45	39.17	1.86	2767	153.73	-	-
3	0.6	0.4	220	45	28.12	2.40	2417	97.83	-	-
4	0.6	0.15	220	45	30.29	1.15	2823	101.42	-	-
5	0.6	0.15	250	45	28.88	0.45	2497	94.67	-	-
6	0.6	0.15	220	45	31.01	2.55	3716	251.73	-	-
7	0.6	0.4	220	0	36.72	2.75	2943	189.45	-	-
8	0.6	0.15	220	0	34.77	2.87	3304	183.06	-	-
9	0.6	0.4	250	0	38.20	0.98	3018	33.38	-	-
10	0.6	0.15	250	0	43.33	1.55	3816	111.38	-	-
11	0.6	0.4	220	0	35.65	1.79	2789	215.70	-	-
12	0.6	0.15	220	0	37.25	12.39	3361	1018	-	-
13	0.6	0.4	220	45	35.05	5.08	2337	423.01	-	-
14	0.6	0.15	220	45	32.43	4.63	2308	293.98	-	-
15	0.6	0.4	250	90	36.86	2.69	2763	140.62	-	-
16	0.6	0.4	250	45	33.94	2.14	1854	124.32	-	-
17	0.6	0.15	250	0	32.71	2.12	2971	225.60	-	-
18	0.6	0.15	250	45	33.09	2.44	2265	209.53	-	-
19	0.3	0.2	220	45	36.09	0.84	2213	200.86	-	-
20	0.3	0.1	220	0	39.24	1.75	3951	460.87	-	-
21	0.3	0.2	220	0	35.00	3.91	2752	281.88	-	-
22	0.3	0.1	220	45	43.73	1.94	3400	167.57	-	-
23	0.3	0.1	220	0	28.94	3.01	2613	232.51	-	-
24	0.3	0.2	220	0	28.66	5.94	2270	421.09	-	-
25	0.3	0.1	220	45	27.08	1.14	1616	56.63	-	-
26	0.3	0.2	220	45	24.88	1.69	1353	100.76	-	-
27	0.3	0.2	250	45	36.50	0.99	2065	195.60	-	-
28	0.3	0.1	250	0	47.47	3.83	5409	508.06	-	-
29	0.3	0.2	250	0	36.81	1.29	2701	219.35	-	-
30	0.3	0.1	250	45	43.54	3.43	3802	364.11	-	-
31	0.3	0.1	250	0	40.29	1.66	3598	340.19	-	-
32	0.3	0.2	250	0	30.75	1.58	2176	147.28	-	-
33	0.3	0.2	250	45	31.37	0.75	1981	73.07	-	-
34	0.3	0.1	250	45	29.68	2.27	1738	275.94	-	-
35	0.4	0.10	220	0	53.96	3.73	3361	400.54	68.99	6.19
36	0.4	0.25	220	0	52.60	2.43	3039	139.61	74.62	3.79
37	0.4	0.10	220	45	45.63	0.72	2859	125.08	55.18	1.04
38	0.4	0.25	220	45	54.61	4.89	3083	61.63	64.96	3.64
39	0.4	0.10	250	0	57.67	3.98	3981	208.52	76.00	6.19
40	0.4	0.25	250	0	59.49	2.27	3762	112.57	78.63	1.75
41	0.4	0.10	250	45	51.27	3.84	3355	121.64	63.24	3.32
42	0.4	0.25	250	45	59.56	2.92	3019	223.98	68.44	3.19
43	0.8	0.20	220	0	56.59	6.51	4032	180.94	70.05	2.52
44	0.8	0.50	220	0	61.24	1.93	3617	168.38	73.53	3.92
45	0.8	0.50	220	45	44.21	3.80	3031	116.94	58.04	4.17
46	0.8	0.50	220	45	43.98	5.56	2852	371.12	53.78	3.92
47	0.8	0.20	250	0	59.30	3.96	4203	154.19	73.00	4.74
48	0.8	0.50	250	0	72.00	7.10	4243	198.44	77.46	5.92
49	0.8	0.20	250	45	51.51	3.67	3396	176.39	-	-
50	0.8	0.50	250	45	46.66	4.45	2973	113.84	-	-

**Table 2:** The values of received parameters of each mechanical property.

Parameter	Mechanical Property		
	$\sigma_{T}$	E	$\sigma_{F}$
*a*	54.39	3425.33	67.20
*b*	2.79	191.17	2.30
*c*	0.04	117.95	−1.56
*d*	1.88	−101.90	1.68
*e*	−4.71	−354.35	−6.84
*f*	0.14	−30.64	−0.52
*g*	0.37	−15.28	−0.15
*h*	−0.22	−76.36	0.07
*j*	−0.34	−20.27	−1.23
*k*	−3.13	−126.04	−1.04
*l*	−0.35	12.57	−0.35

**Figure A1:**
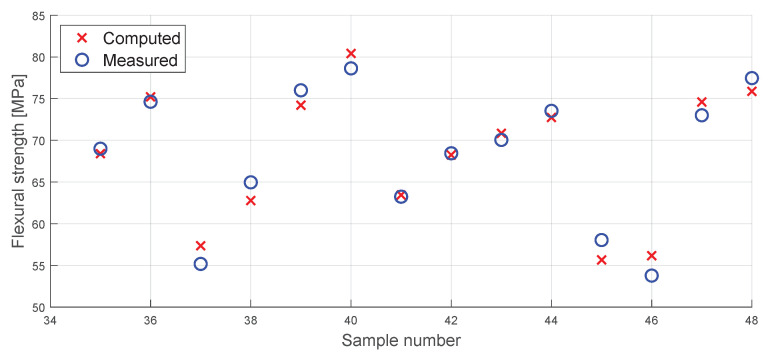
Comparison of the computed and the measured flexural strength.

**Figure A2:**
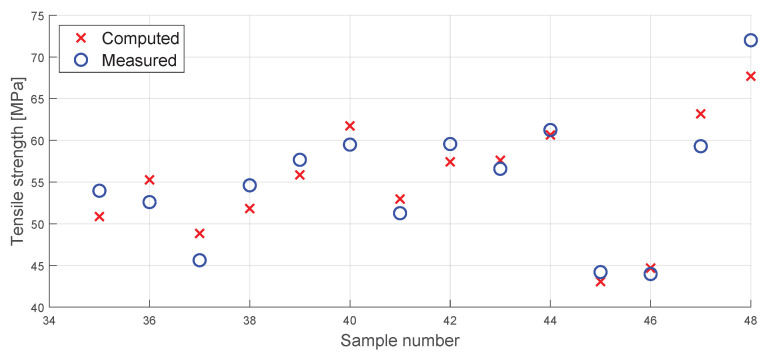
Comparison of the computed and the measured tensile strength.

**Figure A3:**
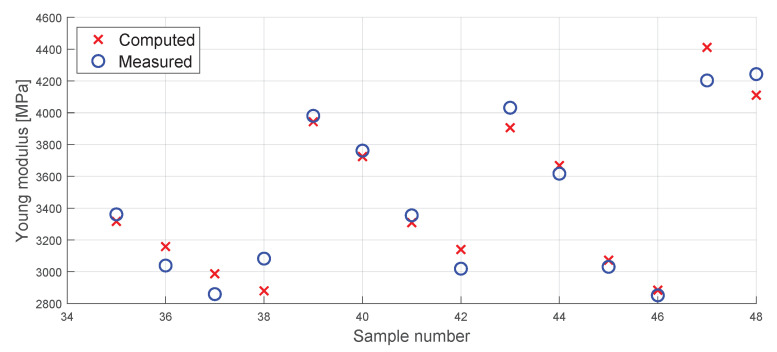
Comparison of the computed and the measured Young’s modulus.

## Data Availability

Data available on request.

## Supplementary Materials

The following supporting information can be downloaded at: www.mdpi.com/xxx/s1.

## Abbreviations

The following abbreviations are used in this manuscript:

*T*	Temperature	(°C)
$D_{N}$	Nozzle diameter	(mm)
$H_{L}$	Layer height	(mm)
α	Infill orientation	(°)
$\sigma_{T}$	Tensile strength	(MPa)
$\sigma_{F}$	Flexural strength	(MPa)
*E*	Young’s modulus	(MPa)
ρ	Pearson coefficient	(-)
*N*	Sizes of the data lines	(-)
*P*	Variables and measured data lines	(-)
μ	Mean	(-)
ω	Standard deviation	(-)
