# Supplementary material for: Glass Fibre-Reinforced Extrusion 3D-Printed Composites: Experimental and Numerical Study of Mechanical Properties
Source: Polymers (Basel). 2024 Jan 11;16(2):0. doi: 10.3390/polym16020212 (PMC11154450; doi:10.3390/polym16020212)
Supplement: Supplementary file 1 [file polymers-16-00212-s001.zip › polymers-2708787-supplementary.pdf]

Supplementary Figure S1 Stress-Strain diagrams for the experiments (multiple curves shows the number of samples)

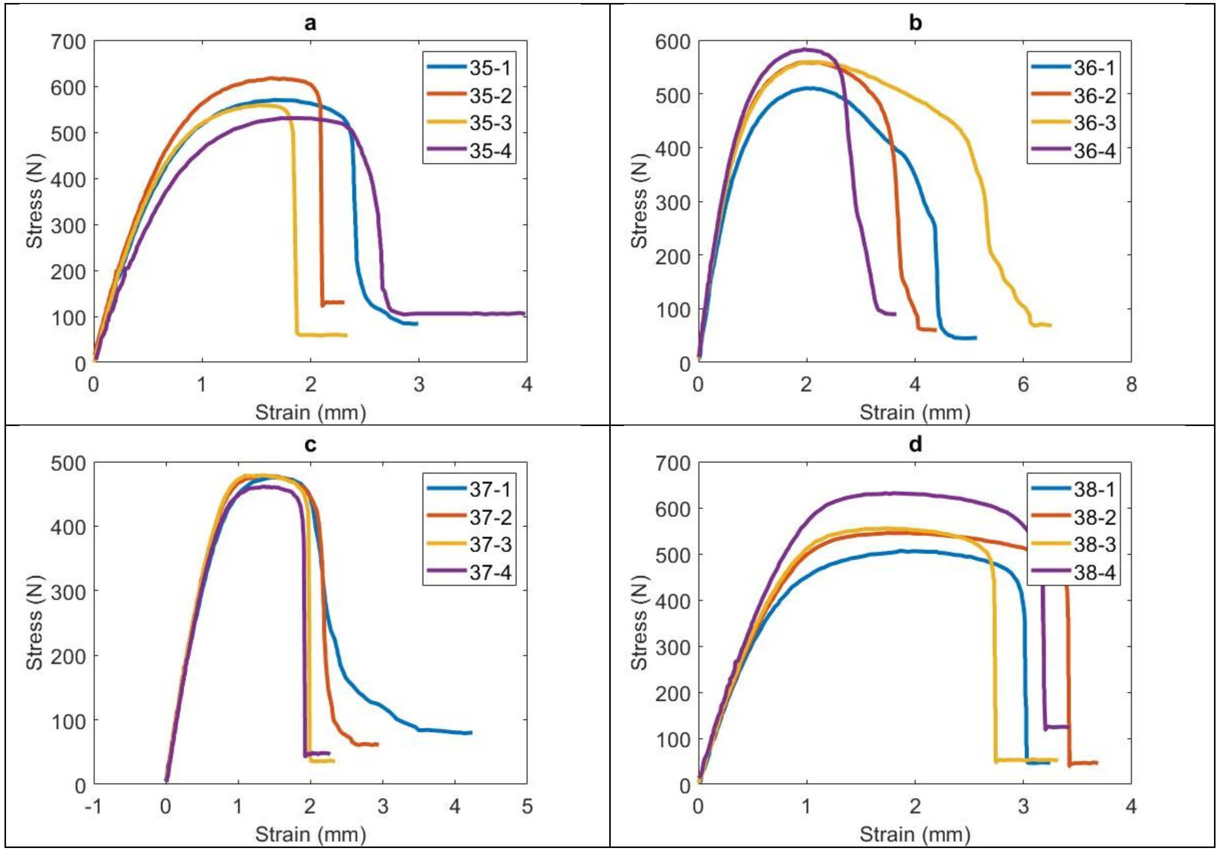

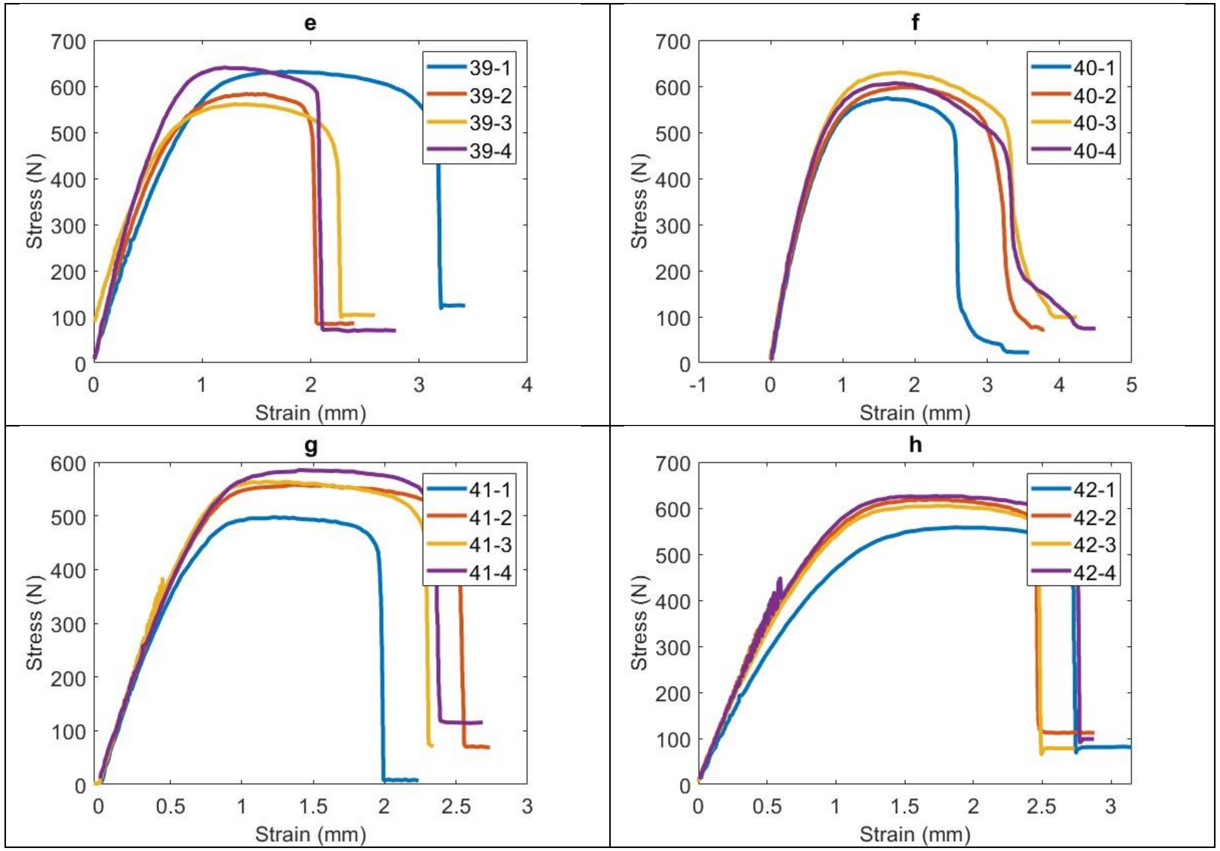
e
39-1
39-2
39-3
39-4
Stress (N)
Strain (mm)
f
40-1
40-2
40-3
40-4
Stress (N)
Strain (mm)
g
41-1
41-2
41-3
41-4
Stress (N)
Strain (mm)
h
42-1
42-2
42-3
42-4
Stress (N)
Strain (mm)

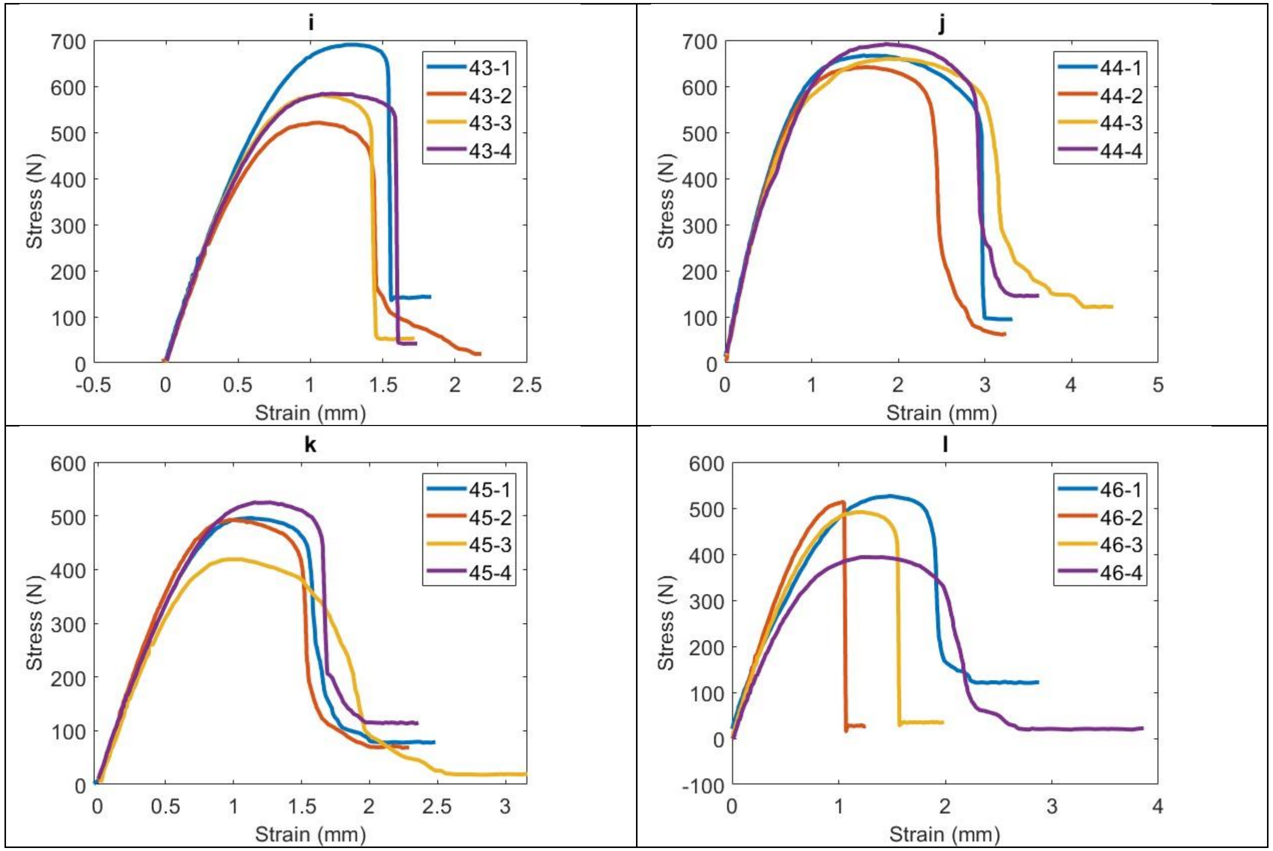
i
43-1
43-2
43-3
43-4
Stress (N)
Strain (mm)
j
44-1
44-2
44-3
44-4
Stress (N)
Strain (mm)
k
45-1
45-2
45-3
45-4
Stress (N)
Strain (mm)
l
46-1
46-2
46-3
46-4
Stress (N)
Strain (mm)

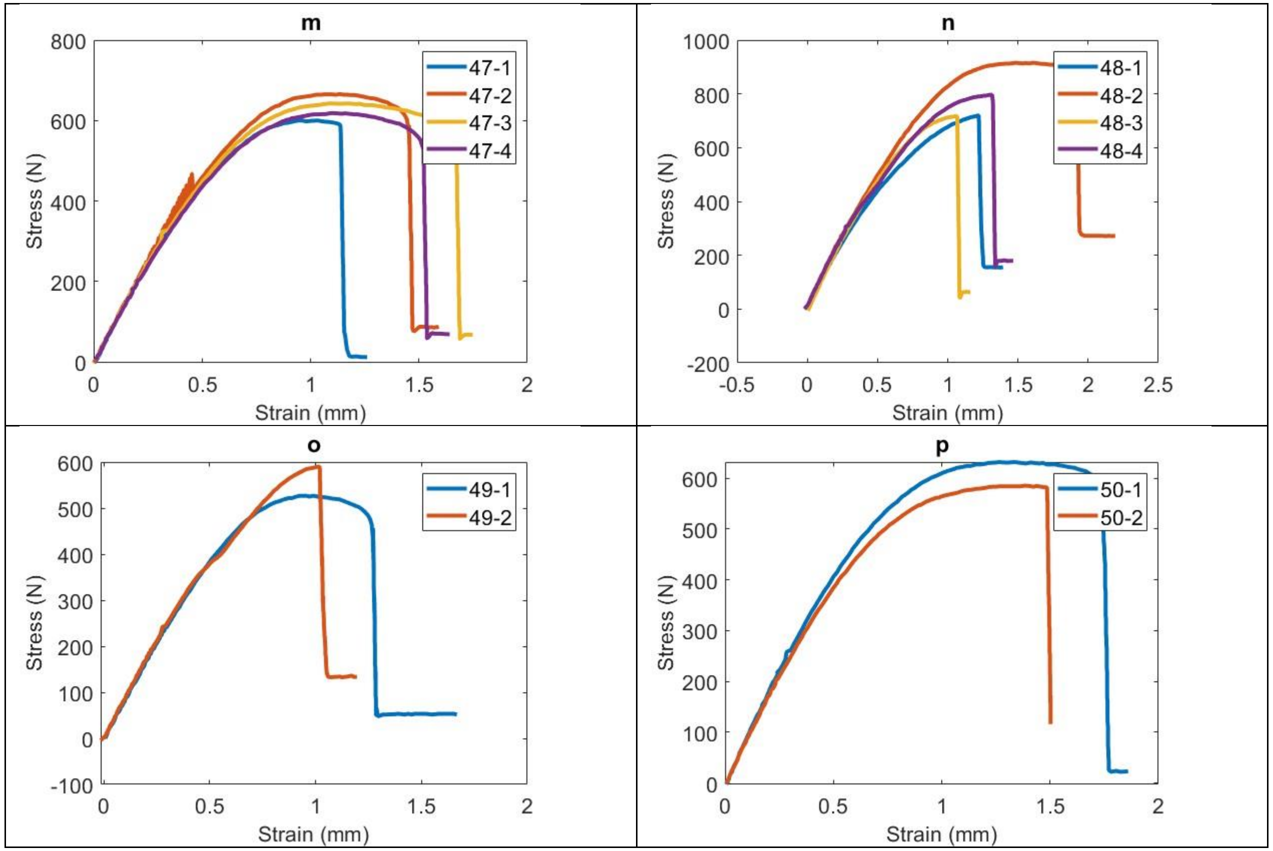
m
n
o
p
Stress (N)
Strain (mm)
47-1
47-2
47-3
47-4
48-1
48-2
48-3
48-4
49-1
49-2
50-1
50-2

Supplementary Table S1 Pearson correlation matrix confidence

	T	D_N	H_L	α	$T \cdot D_N$	$T \cdot H_L$	$T \cdot \alpha$	$D_N \cdot L_H$	$D_N \cdot \alpha$	$H_L \cdot \alpha$	σ_t	E	σ_f
T	[-0.03,0.44]	[0.18,0.59]	[0.16,0.58]	[-0.25,0.25]	[-0.25,0.25]	[-0.25,0.25]	[-0.25,0.25]	[-0.25,0.25]	[-0.25,0.25]	[-0.25,0.25]	[-0.25,0.25]	[-0.25,0.25]	[NaN,NaN]
D_N	[0.15,0.57]	[0.00,0.47]	[-0.24,0.25]	[-0.25,0.25]	[-0.25,0.25]	[-0.25,0.25]	[-0.25,0.25]	[-0.25,0.25]	[-0.25,0.25]	[-0.25,0.25]	[-0.25,0.25]	[-0.25,0.25]	[-0.25,0.25]
H_L	[-0.18,0.31]	[-0.03,0.44]	[0.02,0.48]	[-0.25,0.25]	[-0.25,0.25]	[-0.25,0.25]	[-0.25,0.25]	[-0.25,0.25]	[-0.25,0.25]	[-0.25,0.25]	[NaN,NaN]	[-0.25,0.25]	[-0.25,0.25]
α	[0.42,0.74]	[0.62,0.84]	[0.49,0.78]	[-0.25,0.25]	[-0.25,0.25]	[-0.25,0.25]	[-0.25,0.25]	[-0.25,0.25]	[-0.25,0.25]	[NaN,NaN]	[-0.25,0.25]	[-0.25,0.25]	[-0.25,0.25]
$T \cdot D_N$	[0.10,0.54]	[-0.18,0.31]	[-0.23,0.26]	[-0.25,0.25]	[-0.25,0.25]	[-0.25,0.25]	[-0.25,0.25]	[-0.25,0.25]	[NaN,NaN]	[-0.25,0.25]	[-0.25,0.25]	[-0.25,0.25]	[-0.25,0.25]
$T \cdot H_L$	[-0.24,0.25]	[-0.22,0.28]	[-0.20,0.29]	[-0.25,0.25]	[-0.25,0.25]	[-0.25,0.25]	[-0.25,0.25]	[NaN,NaN]	[-0.25,0.25]	[-0.25,0.25]	[-0.25,0.25]	[-0.25,0.25]	[-0.25,0.25]
$T \cdot \alpha$	[0.07,0.52]	[-0.09,0.39]	[-0.22,0.27]	[-0.25,0.25]	[-0.25,0.25]	[-0.25,0.25]	[NaN,NaN]	[-0.25,0.25]	[-0.25,0.25]	[-0.25,0.25]	[-0.25,0.25]	[-0.25,0.25]	[-0.25,0.25]
$D_N \cdot L_H$	[-0.20,0.29]	[-0.20,0.29]	[-0.20,0.29]	[-0.25,0.25]	[-0.25,0.25]	[NaN,NaN]	[-0.25,0.25]	[-0.25,0.25]	[-0.25,0.25]	[-0.25,0.25]	[-0.25,0.25]	[-0.25,0.25]	[-0.25,0.25]
$D_N \cdot \alpha$	[0.12,0.55]	[0.02,0.48]	[0.22,0.62]	[-0.25,0.25]	[NaN,NaN]	[-0.25,0.25]	[-0.25,0.25]	[-0.25,0.25]	[-0.25,0.25]	[-0.25,0.25]	[-0.25,0.25]	[-0.25,0.25]	[-0.25,0.25]
$H_L \cdot \alpha$	[-0.23,0.26]	[-0.22,0.27]	[-0.20,0.29]	[NaN,NaN]	[-0.25,0.25]	[-0.25,0.25]	[-0.25,0.25]	[-0.25,0.25]	[-0.25,0.25]	[-0.25,0.25]	[-0.25,0.25]	[-0.25,0.25]	[-0.25,0.25]
σ_t	[0.31,0.68]	[0.65,0.86]	[NaN,NaN]	[-0.20,0.29]	[0.22,0.62]	[-0.20,0.29]	[-0.22,0.27]	[-0.20,0.29]	[-0.23,0.26]	[0.49,0.78]	[0.02,0.48]	[-0.24,0.25]	[0.16,0.58]
E	[0.19,0.59]	[NaN,NaN]	[0.65,0.86]	[-0.22,0.27]	[0.02,0.48]	[-0.20,0.29]	[-0.09,0.39]	[-0.22,0.28]	[-0.18,0.31]	[0.62,0.84]	[-0.03,0.44]	[0.00,0.47]	[0.18,0.59]
σ_f	[NaN,NaN]	[0.19,0.59]	[0.31,0.68]	[-0.23,0.26]	[0.12,0.55]	[-0.20,0.29]	[0.07,0.52]	[-0.24,0.25]	[0.10,0.54]	[0.42,0.74]	[-0.18,0.31]	[0.15,0.57]	[-0.03,0.44]

Supplementary Table S2 Specimen table

No.	nozzle type	nozzle diameter (mm)	layer height (mm)	temperature (C°)	shell wall line count (-)	infill orientation (°)
1	MK8	0,6	0,4	220	1	45
2	MK8	0,6	0,4	250	1	45
3	MK8	0,6	0,4	220	1	45
4	MK8	0,6	0,15	220	1	45
5	MK8	0,6	0,15	250	1	45
6	MK8	0,6	0,15	220	5	45
7	MK8	0,6	0,4	220	1	0
8	MK8	0,6	0,15	220	1	0
9	MK8	0,6	0,4	250	1	0
10	MK8	0,6	0,15	250	1	0
11	MK8	0,6	0,4	220	0	0
12	MK8	0,6	0,15	220	0	0
13	MK8	0,6	0,4	220	0	45
14	MK8	0,6	0,15	220	0	45
15	MK8	0,6	0,4	250	0	90
16	MK8	0,6	0,4	250	0	45
17	MK8	0,6	0,15	250	0	0
18	MK8	0,6	0,15	250	0	45
19	MK8	0,3	0,2	220	1	45
20	MK8	0,3	0,1	220	1	0
21	MK8	0,3	0,2	220	1	0
22	MK8	0,3	0,1	220	1	45
23	MK8	0,3	0,1	220	0	0
24	MK8	0,3	0,2	220	0	0

25	MK8	0,3	0,1	220	0	45
26	MK8	0,3	0,2	220	0	45
27	MK8	0,3	0,2	250	1	45
28	MK8	0,3	0,1	250	1	0
29	MK8	0,3	0,2	250	1	0
30	MK8	0,3	0,1	250	1	45
31	MK8	0,3	0,1	250	0	0
32	MK8	0,3	0,2	250	0	0
33	MK8	0,3	0,2	250	0	45
34	MK8	0,3	0,1	250	0	45
35	V6	0,4	0,1	220	0	0
36	V6	0,4	0,25	220	0	0
37	V6	0,4	0,1	220	0	45
38	V6	0,4	0,25	220	0	45
39	V6	0,4	0,1	250	0	0
40	V6	0,4	0,25	250	0	0
41	V6	0,4	0,1	250	0	45
42	V6	0,4	0,25	250	0	45
43	V6	0,8	0,2	220	0	0
44	V6	0,8	0,5	220	0	0
45	V6	0,8	0,5	220	0	45
46	V6	0,8	0,5	220	0	45
47	V6	0,8	0,2	250	0	0
48	V6	0,8	0,5	250	0	0
49	V6	0,8	0,2	250	0	45
50	V6	0,8	0,5	250	0	45

Supplementary Config file: all parameters

generated by SuperSlicer 2.5.59 on 2023-05-15 at 12:40:43 UTC

allow_empty_layers = 0

avoid_crossing_not_first_layer = 1

avoid_crossing_perimeters = 1

avoid_crossing_perimeters_max_detour = 0

bed_custom_model =

bed_custom_texture =

bed_shape = 0x0,400x0,400x400,0x400

bed_temperature = 90

before_layer_gcode = ;BEFORE_LAYER_CHANGE\n;[layer_z]\nG92 E0\n;{if layer_num == 2 }SET_FILAMENT_SENSOR SENSOR=my_sensor ENABLE=1{endif}\n\n

between_objects_gcode =

bottom_fill_pattern = monotonic

bottom_solid_layers = 0

bottom_solid_min_thickness = 0

bridge_acceleration = 0

bridge_angle = 0

bridge_fan_speed = 0

bridge_fill_pattern = rectilinear

bridge_flow_ratio = 80%

bridge_internal_acceleration = 0

bridge_internal_fan_speed = 0

bridge_overlap = 90%

bridge_overlap_min = 80%

bridge_speed = 70%

bridge_speed_internal = 150%

bridge_type = nozzle

bridged_infill_margin = 200%

brim_acceleration = 0

brim_ears = 0

brim_ears_detection_length = 1

brim_ears_max_angle = 125

brim_ears_pattern = concentric

brim_inside_holes = 0

brim_separation = 0

brim_speed = 120%

brim_width = 5

brim_width_interior = 0

chamber_temperature = 0

clip_multipart_objects = 1

color_change_gcode = M600

colorprint_heights =

compatible_printers_condition_cummulative = printer_model=~/.*VC3_.*/;

complete_objects = 0

complete_objects_one_brim = 0

complete_objects_one_skirt = 0

complete_objects_sort = object

cooling_tube_length = 5

cooling_tube_retraction = 91.5

curve_smoothing_angle_concave = 0

curve_smoothing_angle_convex = 0

curve_smoothing_cutoff_dist = 2

curve_smoothing_precision = 0

default_acceleration = 4500

default_filament_profile = "Generic PLA @RatRig"

default_print_profile = 0.20mm NORMAL @RatRig

default_speed = 40

deretract_speed = 45

disable_fan_first_layers = 3

dont_support_bridges = 1

draft_shield = disabled

duplicate_distance = 6

end_filament_gcode = "; Filament-specific end gcode \n;END gcode for filament\n"

end_gcode = END_PRINT\n

enforce_full_fill_volume = 0

enforce_retract_first_layer = 1

ensure_vertical_shell_thickness = 1

exact_last_layer_height = 0

external_infill_margin = 150%

external_perimeter_acceleration = 10%

external_perimeter_cut_corners = 0%

external_perimeter_extrusion_spacing =

external_perimeter_extrusion_width = 100%

external_perimeter_fan_speed = 0

external_perimeter_overlap = 100%

external_perimeter_speed = 80%

external_perimeters_first = 0

external_perimeters_hole = 1

external_perimeters_nothole = 1

external_perimeters_vase = 0

extra_loading_move = -2

extra_perimeters = 0

extra_perimeters_odd_layers = 0

extra_perimeters_overhangs = 0

extruder_clearance_height = 25

extruder_clearance_radius = 45

extruder_colour = ""

extruder_fan_offset = 0%

extruder_offset = 0x0

extruder_temperature_offset = 0

extrusion_axis = E

extrusion_multiplier = 0.92

extrusion_spacing = 0

extrusion_width =

fan_always_on = 0

fan_below_layer_time = 10

fan_kickstart = 0

fan_percentage = 0

fan_speedup_overhangs = 1

fan_speedup_time = 0

feature_gcode =

filament_colour = #3A80CA

filament_cooling_final_speed = 3.4

filament_cooling_initial_speed = 2.2

filament_cooling_moves = 4

filament_cooling_zone_pause = 0

filament_cost = 60

filament_custom_variables = ""

filament_density = 1.24

filament_deretract_speed = nil

filament_diameter = 1.75

filament_dip_extraction_speed = 70

filament_dip_insertion_speed = 33

filament_enable_toolchange_part_fan = 0

filament_enable_toolchange_temp = 0

filament_load_time = 0

filament_loading_speed = 28

filament_loading_speed_start = 3

filament_max_overlap = 100%

filament_max_speed = 0

filament_max_volumetric_speed = 4

filament_max_wipe_tower_speed = 0

filament_melt_zone_pause = 0

filament_minimal_purge_on_wipe_tower = 15

filament_notes = ""

filament_ramming_parameters = "120 100 6.6 6.8 7.2 7.6 7.9 8.2 8.7 9.4 9.9 10.0| 0.05 6.6 0.45 6.8 0.95 7.8 1.45 8.3 1.95 9.7 2.45 10 2.95 7.6 3.45 7.6 3.95 7.6 4.45 7.6 4.95 7.6"

filament_retract_before_travel = nil

filament_retract_before_wipe = nil

filament_retract_layer_change = nil

filament_retract_length = 2

filament_retract_lift = nil

filament_retract_lift_above = nil

filament_retract_lift_below = nil

filament_retract_restart_extra = nil

filament_retract_speed = nil

filament_seam_gap = nil

filament_settings_id = "Esun ePA12 @RatRig - Copy20230313"

filament_shrink = 100%

filament_skinnydip_distance = 31

filament_soluble = 0

filament_spool_weight = 0

filament_toolchange_delay = 0

filament_toolchange_part_fan_speed = 50

filament_toolchange_temp = 200

filament_type = NYLON

filament_unload_time = 0

filament_unloading_speed = 90

filament_unloading_speed_start = 100

filament_use_fast_skinnydip = 0

filament_use_skinnydip = 0

filament_vendor = Esun

filament_wipe = 1

filament_wipe_advanced_pigment = 0.5

filament_wipe_extra_perimeter = nil

filament_wipe_inside_depth = nil

filament_wipe_inside_end = nil

filament_wipe_inside_start = nil

filament_wipe_only_crossing = nil

filament_wipe_speed = nil

fill_angle = 0

fill_angle_increment = 0

fill_density = 100%

fill_pattern = rectilinear

fill_smooth_distribution = 10%

fill_smooth_width = 50%

fill_top_flow_ratio = 100%

first_layer_acceleration = 10%

first_layer_acceleration_over_raft = 0

first_layer_bed_temperature = 90

first_layer_extrusion_spacing =

first_layer_extrusion_width = 100%

first_layer_flow_ratio = 100%

first_layer_height = 0.3

first_layer_infill_speed = 0

first_layer_min_speed = 20

first_layer_size_compensation = -0.1

first_layer_size_compensation_layers = 1

first_layer_speed = 35%

first_layer_speed_over_raft = 30

first_layer_temperature = 285

full_fan_speed_layer = 0

fuzzy_skin = none

fuzzy_skin_point_dist = 200%

fuzzy_skin_thickness = 150%

gap_fill_acceleration = 0

gap_fill_enabled = 1

gap_fill_extension = 0

gap_fill_flow_match_perimeter = 0%

gap_fill_last = 0

gap_fill_max_width = 0

gap_fill_min_area = 100%

gap_fill_min_length = 0

gap_fill_min_width = 0

gap_fill_overlap = 100%

gap_fill_speed = 80%

gcode_comments = 0

gcode_filename_illegal_char = [<>:"/\\\\|?*]

gcode_flavor = klipper

gcode_label_objects = 1

gcode_precision_e = 4

gcode_precision_xyz = 3

gcode_resolution = 0

gcode_substitutions =

high_current_on_filament_swap = 0

hole_size_compensation = 0

hole_size_threshold = 100

hole_to_polyhole = 0

hole_to_polyhole_threshold = 0.01

hole_to_polyhole_twisted = 1

host_type = octoprint

infill_acceleration = 4500

infill_anchor = 600%

infill_anchor_max = 5

infill_connection = connected

infill_connection_bottom = connected

infill_connection_bridge = connected

infill_connection_solid = connected

infill_connection_top = connected

infill_dense = 0

infill_dense_algo = autoenlarged

infill_every_layers = 1

infill_extruder = 1

infill_extrusion_spacing =

infill_extrusion_width = 100%

infill_first = 0

infill_only_where_needed = 0

infill_overlap = 18%

infill_speed = 250%

inherits_cummulative = "0.20mm NORMAL @RatRig";"Esun ePA-CF @RatRig";"RatRig V-Core-3-400"

init_z_rotate = 0

interface_shells = 0

ironing = 0

ironing_acceleration = 0

ironing_angle = -1

ironing_flowrate = 15%

ironing_spacing = 0.1

ironing_speed = 15

ironing_type = top

layer_gcode = ;AFTER_LAYER_CHANGE\n;[layer_z]\n

layer_height = 0.3

lift_min = 0

machine_limits_usage = time_estimate_only

machine_max_acceleration_e = 5000

machine_max_acceleration_extruding = 9000

machine_max_acceleration_retracting = 9000

machine_max_acceleration_travel = 9000,1250

machine_max_acceleration_x = 9000

machine_max_acceleration_y = 9000

machine_max_acceleration_z = 100

machine_max_feedrate_e = 60

machine_max_feedrate_x = 500

machine_max_feedrate_y = 500

machine_max_feedrate_z = 10

machine_max_jerk_e = 5

machine_max_jerk_x = 5

machine_max_jerk_y = 5

machine_max_jerk_z = 0.4

machine_min_extruding_rate = 0

machine_min_travel_rate = 0

max_fan_speed = 0

max_gcode_per_second = 1500

max_layer_height = 0.5

max_print_height = 400

max_print_speed = 500

max_speed_reduction = 90%

max_volumetric_speed = 12

milling_after_z = 200%

milling_diameter =

milling_extra_size = 150%

milling_post_process = 0

milling_speed = 30

milling_toolchange_end_gcode =

milling_toolchange_start_gcode =

milling_z_lift =

min_bead_width = 85%

min_fan_speed = 0

min_feature_size = 0.1

min_layer_height = 0.2

min_length = 0.035

min_print_speed = 15

min_skirt_length = 20

min_width_top_surface = 100%

mmu_segmented_region_max_width = 0

model_precision = 0.0001

no_perimeter_unsupported_algo = noperi

notes =

nozzle_diameter = 0.8

only_one_perimeter_first_layer = 0

only_one_perimeter_top = 0

only_one_perimeter_top_other_algo = 0

only_retract_when_crossing_perimeters = 0

ooze_prevention = 0

output_filename_format = {input_filename_base}_{layer_height}mm_{filament_type[0]}_{print_time}.gcode

over_bridge_flow_ratio = 100%

overhangs_acceleration = 0

overhangs_reverse = 0

overhangs_reverse_threshold = 250%

overhangs_speed = 100%

overhangs_width = 75%

overhangs_width_speed = 55%

parking_pos_retraction = 92

pause_print_gcode = M601

perimeter_acceleration = 4500

perimeter_bonding = 0%

perimeter_extruder = 1

perimeter_extrusion_spacing =

perimeter_extrusion_width = 100%

perimeter_generator = classic

perimeter_loop = 0

perimeter_loop_seam = rear

perimeter_overlap = 100%

perimeter_round_corners = 0

perimeter_speed = 120%

perimeters = 0

physical_printer_settings_id =

post_process =

print_custom_variables =

print_extrusion_multiplier = 100%

print_host =

print_retract_length = -1

print_retract_lift = -1

print_settings_id = NYLON RATRIG20230110probatest04and08nozzle

print_temperature = 0

printer_custom_variables =

printer_model = VC3_400

printer_notes = Don't remove the following keywords! These keywords are used in the "compatible printer" condition of the print and filament profiles to link the particular print and filament profiles to this printer profile.\nPRINTER_VENDOR_RatRig\nPRINTER_MODEL_VCORE\nPRINTER_HAS_Directextruder\nE3DV6

printer_settings_id = NYLON0.8mmRatRig V-Core-3-400first

printer_technology = FFF

printer_variant = 0.4

printer_vendor =

printhost_apikey =

printhost_cafile =

printhost_port =

raft_contact_distance = 0.1

raft_expansion = 3

raft_first_layer_density = 90%

raft_first_layer_expansion = 6

raft_interface_layer_height = 0

raft_layer_height = 0

raft_layers = 0

remaining_times = 1

remaining_times_type = m73m117

resolution = 0.0125

resolution_internal = 0.2

retract_before_travel = 3

retract_before_wipe = 0%

retract_layer_change = 0

retract_length = 1

retract_length_toolchange = 1

retract_lift = 0.2

retract_lift_above = 0

retract_lift_below = 0

retract_lift_first_layer = 1

retract_lift_top = "All surfaces"

retract_restart_extra = 0

retract_restart_extra_toolchange = 0

retract_speed = 45

seam_angle_cost = 80%

seam_gap = 15%

seam_position = cost

seam_travel_cost = 20%

silent_mode = 0

single_extruder_multi_material = 0

single_extruder_multi_material_priming = 1

skirt_brim = 0

skirt_distance = 20

skirt_distance_from_brim = 1

skirt_extrusion_width = 110%

skirt_height = 1

skirts = 1

slice_closing_radius = 0.049

slicing_mode = regular

slowdown_below_layer_time = 10

small_perimeter_max_length = 20

small_perimeter_min_length = 6

small_perimeter_speed = 80%

solid_fill_pattern = rectilinear

solid_infill_acceleration = 0

solid_infill_below_area = 0

solid_infill_every_layers = 0

solid_infill_extruder = 1

solid_infill_extrusion_spacing =

solid_infill_extrusion_width = 100%

solid_infill_overlap = 100%

solid_infill_speed = 80%

solid_over_perimeters = 2

spiral_vase = 0

standby_temperature_delta = -5

start_filament_gcode = "; Filament gcode\nSET_GCODE_OFFSET Z=0.0\n\n{if nozzle_diameter[0]==0.4} SET_PRESSURE_ADVANCE ADVANCE=0.045{elsif nozzle_diameter[0]==0.6}SET_PRESSURE_ADVANCE ADVANCE=0.02{endif}\n\n"

start_gcode = START_PRINT EXTRUDER_TEMP=[first_layer_temperature] BED_TEMP=[first_layer_bed_temperature]

start_gcode_manual = 0

support_material = 0

support_material_acceleration = 0

support_material_angle = 0

support_material_angle_height = 0

support_material_auto = 0

support_material_bottom_contact_distance = 0.2

support_material_bottom_interface_layers = -1

support_material_buildplate_only = 1

support_material_closing_radius = 2

support_material_contact_distance = 50%

support_material_contact_distance_type = filament

support_material_enforce_layers = 0

support_material_extruder = 0

support_material_extrusion_width = 80%

support_material_interface_acceleration = 0

support_material_interface_angle = 90

support_material_interface_angle_increment = 0

support_material_interface_contact_loops = 0

support_material_interface_extruder = 0

support_material_interface_fan_speed = -1

support_material_interface_layer_height = 0

support_material_interface_layers = 2

support_material_interface_pattern = rectilinear

support_material_interface_spacing = 0.2

support_material_interface_speed = 100%

support_material_layer_height = 0

support_material_pattern = rectilinear

support_material_spacing = 2

support_material_speed = 80%

support_material_style = snug

support_material_synchronize_layers = 0

support_material_threshold = 65

support_material_with_sheath = 0

support_material_xy_spacing = 60%

temperature = 285

template_custom_gcode =

thin_perimeters = 80%

thin_perimeters_all = 20%

thin_walls = 1

thin_walls_acceleration = 0

thin_walls_merge = 1

thin_walls_min_width = 33%

thin_walls_overlap = 50%

thin_walls_speed = 100%

threads = 8

thumbnails = 32x32,400x300

thumbnails_color = #018aff

thumbnails_custom_color = 0

thumbnails_end_file = 0

thumbnails_format = PNG

thumbnails_with_bed = 1

time_cost = 0

time_estimation_compensation = 100%

time_start_gcode = 20

time_toolchange = 30

tool_name = ""

toolchange_gcode =

top_fan_speed = 0

top_fill_pattern = monotonic

top_infill_extrusion_spacing =

top_infill_extrusion_width = 100%

top_solid_infill_acceleration = 0

top_solid_infill_speed = 60%

top_solid_layers = 0

top_solid_min_thickness = 0

travel_acceleration = 4500

travel_deceleration_use_target = 1

travel_speed = 400

travel_speed_z = 0

use_firmware_retraction = 0

use_relative_e_distances = 1

use_volumetric_e = 0

variable_layer_height = 1

wall_add_middle_threshold = 75%

wall_distribution_count = 1

wall_split_middle_threshold = 50%

wall_transition_angle = 10

wall_transition_filter_deviation = 25%

wall_transition_length = 0.4

wipe = 0

wipe_advanced = 0

wipe_advanced_algo = linear

wipe_advanced_multiplier = 60

wipe_advanced_nozzle_melted_volume = 120

wipe_extra_perimeter = 0

wipe_inside_depth = 50%

wipe_inside_end = 1

wipe_inside_start = 0

wipe_into_infill = 0

wipe_into_objects = 0

wipe_only_crossing = 1

wipe_speed = 0

wipe_tower = 0

wipe_tower_bridging = 10

wipe_tower_brim_width = 2

wipe_tower_no_sparse_layers = 0

wipe_tower_rotation_angle = 0

wipe_tower_width = 60

wipe_tower_x = 170

wipe_tower_y = 140

wiping_volumes_extruders = 70,70

wiping_volumes_matrix = 0

xy_inner_size_compensation = 0

xy_size_compensation = 0

z_offset = -0.05

z_step = 0.005
